# Long-Term Emotional Impact of the COVID-19 Pandemic and Barriers and Facilitators to Digital Mental Health Tools in Long-Term Care Workers: Qualitative Study

**DOI:** 10.2196/47546

**Published:** 2024-05-29

**Authors:** Leticia González-Spinoglio, Anna Monistrol-Mula, Cecilia Vindrola-Padros, Salvatore Aguilar-Ortiz, Bernat Carreras, Josep Maria Haro, Mireia Felez-Nobrega

**Affiliations:** 1 Research and Development Unit, Parc Sanitari Sant Joan de Déu Institut Sant Joan de Déu Barcelona Spain; 2 Centre for Biomedical Research on Mental Health Madrid Spain; 3 Department of Targeted Intervention University College London London United Kingdom; 4 Department of Psychiatry and Psychology Institute of Neuroscience, Hospital Clínic Barcelona Spain; 5 Departament de Medicina Universitat de Barcelona Barcelona Spain

**Keywords:** COVID-19 pandemic, digital technology, health care professionals, long-term care, mental health, well-being, digital mental health, digital mental health interventions, mobile phone

## Abstract

**Background:**

The overall pandemic created enormous pressure on long-term care workers (LTCWs), making them particularly vulnerable to mental disorders. Despite this, most of the available evidence on professional well-being during COVID-19 has exclusively focused on frontline health care workers.

**Objective:**

This study aimed to identify the long-term psychological needs of LTCWs derived from the COVID-19 pandemic and to explore barriers and facilitators related to digital mental health tools. This is part of a project that seeks to develop a digital mental health intervention to reduce psychological distress in this population group.

**Methods:**

We performed a qualitative study with a rapid research approach. Participants were LTCWs of the autonomous community of Catalonia. We conducted 30 semistructured interviews between April and September 2022. We used a qualitative content analysis method with an inductive-deductive approach.

**Results:**

The period of the pandemic with the highest mental health burden was the COVID-19 outbreak, with almost all workers having experienced some form of emotional distress. Emotional distress persisted over time in more than half of the participants, with fatigue and nervousness being the main emotions expressed at the time of the interview. High workload, the feeling that pandemic times are not over, and poor working conditions that have remained since then have been the most frequently expressed determinants of such emotions. Potential barriers and facilitators to engagement with digital tools were also identified in terms of previous experience and beliefs of the target population, possibilities for the integration of a digital tool into daily life, preferences regarding the level of guidance, the possibility of social connectedness through the tool, and privacy and confidentiality. The identified factors may become especially relevant in the context of the pandemic remission phase.

**Conclusions:**

More than 2 years after the pandemic outbreak, emotional distress is still relevant. The persistent burden of psychological distress points to a need for institutions to take action to improve working conditions and promote employees’ well-being. Considering factors that act as barriers and facilitators for the use of digital mental health tools, it is important to develop tailored tools that could offer valuable support to this population during and after a pandemic.

## Introduction

Long-term care facilities, such as nursing homes and other assisted living facilities, have been hit particularly hard by the COVID-19 pandemic. By February 2021, approximately 41% of the global COVID-19-associated mortality occurred in long-term care residents [1]. In Spain, the COVID-19 outbreak entailed especially tragic consequences, mainly caused by the precariousness of these care systems [2]. According to data from the Spanish Ministry of Health, 27,359 long-term residents died between April 6 and June 20, 2020, which represents approximately 70% of the total COVID-19 deaths. Long-term care workers (LTCWs) have been constantly exposed to anguish and death, have witnessed the social isolation and loneliness of residents due to health restrictions, and have experienced a drastic increase in their working demands [3-5]. Consequently, the mental health and well-being of this population group have been compromised, with clinically relevant levels of stress, anxiety, and depression increasing to nearly 60% from March 2020 to June 2020 [4].

As we approach the third year of the pandemic, health and care workers continue to respond to uncertainties, potential new waves, and the long-term effects of COVID-19. Importantly, recent evidence highlights increased concerns for burnout in this population [6], which can ultimately jeopardize the quality of health care as well as patient safety [7]. While mounting evidence has assessed the mental health experiences, views, and needs of care professionals during the early stages of the pandemic, there is scarce knowledge about the long-term emotional impact. This information is crucial to develop tailored interventions in response to their emotional demands.

Digital mental health tools (DMHTs) are a promising strategy to mitigate psychological consequences in the context of a long-lasting pandemic since they can be delivered remotely, avoiding the risk of transmission of the infection [8]. Yet, low levels of user engagement have been recognized as important barriers that may compromise the efficacy and scalability of interventions [9]. Increasing (prepandemic) evidence is beginning to unravel key factors that influence user engagement and the effectiveness of engagement strategies. For instance, positive prior experiences with mental health services and technology, digital literacy, the credibility of content, guidance within the intervention, increased social connectedness, a greater number of engagement features, reminders, and tailored feedback have been identified as potential facilitators for promoting engagement or effectiveness of digital interventions [10-13]. Nonetheless, these are general features reported in studies that included a wide range of different populations (eg, refugees, general population, people who are homeless, and people with a diagnosis of mental disorders), and it is well recognized that understanding the settings and population’s needs (eg, COVID-19 pandemic and long-term care facility characteristics) is a key factor for user engagement and intervention implementation success [14].

In this context, this study aimed to identify the long-term psychological needs of LTCWs as well as explore barriers and facilitators in relation to DMHTs. These findings will be used to inform and guide the development of a digital intervention to reduce psychological distress in this population group. The effectiveness of the psychological digital intervention will be tested through a randomized clinical trial (ClinicalTrials.gov NCT05526235).

## Methods

### Study Design, Sampling, and Participants

This qualitative study was conducted with a phenomenological orientation, in which we used a rapid research approach. In time-sensitive contexts, such as a pandemic, rapid appraisals allow us to collect and analyze data in a targeted and iterative way within limited time frames and, consequently, adapt and design expedient interventions for improving health care [15]. For reporting purposes, we adhered to the COREQ (Consolidated Criteria for Reporting Qualitative Research) checklist [16].

Semistructured interviews were conducted with workers from long-term care facilities (including nursing homes and other long-term care facilities), who were on duty during the pandemic in the autonomous community of Catalonia. For the recruitment of participants, we contacted several long-term care facilities and presented the project to each representative. Once the facility agreed to participate, the managerial staff shared the study information sheet among their employees (via their usual official means of communication). When needed, the researcher conducted informative talks (in person or via teleconference) with LTCWs in order to disseminate the study and resolve doubts, if any. A total of 6 long-term care facilities from the metropolitan area of Barcelona participated in the study.

### Ethical Considerations

Ethics approval was provided by the Fundació Sant Joan de Déu Ethics Committee, Barcelona, Spain (PIC 214-21). Participants were fully informed about the objectives and procedures of the study prior to signing the informed consent. The data set was pseudoanonymized, and personal data were stored separately and securely on institution servers.

### Data Collection

Interviews were conducted between April and September 2022. They were individual, by videoconference, and lasted between 25 and 40 minutes. The interview guide (provided in Multimedia Appendix 1) was reviewed by 2 experts in quality-of-life research to ensure its adequacy, and it was then tested with the first 2 interviews. No adaptations had to be made. The interview guide related to the digital mental health section was based on the framework developed by Borghouts et al [11]. Participants were given the option of conducting the interview in Spanish or Catalan, according to their preferences. Two researchers (AM-M and LG-S) conducted the interviews. Both are female PhD candidates. AM-M conducted the interviews in Catalan (8/30, 27% interviews), and LG-S conducted the interviews in Spanish (22/30, 73%). Both interviewers were previously trained in qualitative interview methods. Participants were informed of the interviewers’ educational and professional background, and no working or personal relationship existed between participants and interviewers. All interviews were audio recorded, and the main points were documented in real-time notes. At the conclusion of each interview, the emerging findings were synthesized using a RREAL (Rapid Research Evaluation and Appraisal Lab) Sheet (provided in Multimedia Appendix 2). The RREAL Sheet is a flexible working document, normally presented as a table, designed for the collection, synthesis, reporting, and analysis of data [17]. It enhances familiarization with the data and facilitates analysis while data collection is still ongoing [15,17]. First, a table organized by categories was designed using the interview script as a guide, and we piloted or amended this RREAL Sheet during initial data collection. Then, after each interview, we registered the key data obtained using real-time notes. In instances where pertinent details were absent, we referred to the audio recordings for further clarification. Importantly, the categories represented in the table were modified whenever considered necessary during data collection. Finally, the RREAL Sheet was used to guide an in-depth analysis [17]. The sample size was determined by thematic saturation [18].

### Data Analysis

We used a qualitative content analysis method with an inductive-deductive approach. We performed a 1-page table that summarized the core components registered on the RREAL Sheet to identify key themes [17]. Once key themes and specific issues (subthemes) were recognized, we selected quotes from the interviews that could exemplify these themes. Investigator triangulation ensured that the themes reflected the full range and depth of the data.

## Results

### Sample Characteristics

A total of 30 LTCWs participated in the study. The mean age was 44 (SD 11.4) years, most of our sample were women (n=26, 87%), and one-third (n=10, 33%) were from foreign nationalities. The vast majority of the participants were geriatric nursing assistants (n=17, 57%), followed by nurses (n=5, 17%). A more detailed description of the sociodemographic and occupational characteristics of the sample can be found in Table 1.

**Table 1:** Sample characteristics (N=30).

Variables		Values, n (%)
**Age range (years)**		
	<35	7 (23)
	35-49	10 (33)
	≥50	13 (43)
**Gender**		
	Woman	26 (87)
	Man	4 (13)
**Nationality**		
	Spanish	20 (67)
	Foreign	10 (33)
**Professional role**		
	Nurse	5 (17)
	Psychologist	2 (7)
	Geriatric nursing assistant	17 (57)
	Physiotherapist	2 (7)
	Others^a^	4 (13)
**Years in service**		
	<5	7 (23)
	5-15	13 (43)
	>15	10 (33)
**Workday**		
	Full time	21 (70)
	Part time	9 (30)

^a^Director (n=1), occupational therapist (n=1), social worker (n=1), and cleaning staff (n=1).

### Impact of COVID-19 on LTCWs’ Mental Health

Almost all participants identified the COVID-19 outbreak as the worst period for their mental health and reported having experienced emotional distress during this stage. The main emotions expressed were fear, helplessness, abandonment, loneliness, and sadness. Less frequently expressed emotions also included frustration, anger, uncertainty, and exhaustion. Five themes emerged as the main perceived determinants of these emotions:

1. Unexpected and sudden nature of the crisis: Most participants expressed problems related to facing something new and completely unknown for which they were not prepared and its consequences. The reported main causes of distress were lack of information and knowledge, shortage of materials and human resources, problems with institutional organization, and the constant change of protocols and having to readjust to them.
2. High exposure to emotional anguish and death: Half of the participants referred to high exposure to death and emotional anguish. They mainly mentioned the high mortality among residents and the helplessness of witnessing their isolation due to suspended family visits and reduced interactions resulting from health restrictions. Several participants also referred to the emotional anguish caused by the deaths of colleagues, family members, or friends as well as seeing the anguish in their colleagues on a daily basis.
3. Fear of infection: Almost half of the participants mentioned the strong fear of infecting themselves, residents, and family members as well as the potential consequences of infection (eg, the severity of the disease and death).
4. Moral distress: Many workers reported feelings of hopelessness, expressing that nothing they did for the residents in terms of care was enough. This was accompanied, in many cases, by a sense of loss of control of the situation during their caregiving duties.
5. Lack of support: Many workers referred to a lack of recognition and support from authorities (both at the workplace and from the overall health authorities).

Textbox 1 provides an overview of these themes with example quotes for supporting data.

**Main determinants of emotional distress during the outbreak and example quotes.**

**Unexpected and sudden nature of the crisis**

- “We were not prepared; it came from one moment to the next.” [REPICAL (Reducing the psychosocial impact of the Covid-19 pandemic on workers of assisted living facilities)-007]
- “I also had to guide others in my charge, about things that I did not know either.” [REPICAL-004]
- “The protocols that we received were not clear...We were all in a drifting boat.” [REPICAL-008]
- “Overnight we were alone with co-workers. We were very lost; we did not have personal protective equipment and we did not know how to act. There were 4 of us and we managed as best as we could.” [REPICAL-026]

**High exposure to emotional anguish and death**

- “The worst thing I experienced was seeing so many residents die in such a short time, people who were fine and the next day they were gone.” [REPICAL-019]
- “We were not aware of the magnitude of this until residents began to die, they began to die one after the other, 4 or 5 per day (when before maybe one died every 2 months).” [REPICAL-026]
- “Seeing the loneliness in the residents affected me a lot, seeing that they were going to die alone, that they were not able to be with their family. It also affected me to see my co-workers, the most cheerful ones, defeated.” [REPICAL-021]
- “I felt so helpless seeing the isolated residents. We were asked not to have contact with them, but they needed someone to hold their hand.” [REPICAL-030]

**Moral distress**

- “We had to take measures that, while necessary, we knew were not good for the residents.” [REPICAL-003]
- “No matter what we did, people kept dying.” [REPICAL-004]
- “We saw that they were unwell and we could not help them...we wanted to address everything, but we could not.” [REPICAL-028]

**Fear of infection**

- “I even moved and lived in the residence for a while, so as not to infect the residents.” [REPICAL-019]
- “I had to isolate myself at home and I could not be with my child because I was afraid of infecting him. I also used to think: if something happens to me and my husband, who will take care of my child?” [REPICAL-023]

**Lack of support**

- “What I felt most was abandonment.” [REPICAL-002]
- “Feeling of helplessness of not being able to do anything and not having help from anyone.” [REPICAL-010]

In relation to their current emotional state, over half of the participants expressed some type of persistence of emotional distress over time. Among all participants, a minority reported maintaining intense distress, and a significant portion expressed only some improvement since the outbreak. The emotions most often mentioned were fatigue and nervousness, followed by anger and sadness. Three themes emerged as the main determinants of the persistent emotional distress:

1. High workload: Long shifts due to staff shortages and coping with postcrisis backlog.
2. Pandemic times are not over: Fatigue and residual discomfort from being under stress for so long as well as the feeling of not being able to completely move forward from COVID-19 due to the nature of their work.
3. Still poor working conditions: Although the pandemic clearly highlighted the precariousness of these health care systems, some participants claimed that no measures have been taken to address this situation and that, as a result, working conditions remain poor. Relatedly, LTCWs felt that there is an overall failure in learning from the lessons of the COVID-19 pandemic.

Textbox 2 provides an overview of these themes with example quotes for supporting data.

On the other hand, among those participants who expressed a noticeable improvement over time in their emotional discomfort, hope and relief were the most commonly mentioned emotions. Improvement was associated with an increased sense of control over the situation, confidence in vaccines, decreased measures of isolation, and the feeling of regaining prepandemic working dynamics.

**Main determinants of persistent emotional distress and example quotes.**

**High workload**

- “I am overwhelmed. In 2020, they increased my working hours. I was told it would only be during the state of emergency, but I am still not doing my usual schedule. In addition, people have left the workforce, and they have not hired more. I do not have as much time as I would like for other things.” [REPICAL-004]
- “Many projects were stopped because of the pandemic and now they have to be ready overnight. The workload is unreal.” [REPICAL-001]
- “Now we are facing our patients’ pathologies that were neglected during these two years. I am exhausted.” [REPICAL-005]

**Pandemic times are not over**

- “I am tired and burned out from dragging on for so long in tension, needing it to be over and it will not end.” [REPICAL-003]
- “Everyone has moved on, but I am still there: with the masks, the protective equipment, the heat.” [REPICAL-031]
- “During the crisis, we were alert at night in case a co-worker called us for help, and we always called each other; now I keep waking up even if they do not call me.” [REPICAL-026]

**Still poor working conditions**

- “With the pandemic, deficits that already existed in our field have come to light, and the thing is that nothing has changed, it’s just going back to the old ways. Nothing has been learned.” [REPICAL-002]

### Digital Mental Health Tools

#### Previous Experience and Beliefs

Almost all participants reported having no previous experience with DMHTs and emphasized their lack of confidence in their own digital skills. Nonetheless, the vast majority reported believing that such tools could be helpful in improving their mental health. The minority who reported not having confidence in digital tools mainly referred to their preference for personal, face-to-face contact.

#### Integration Into Daily Life

When participants were asked about what characteristics a DMHT should have to make it easy for them to integrate it into their daily lives, the majority of participants referred to the importance of accessibility: “easy to use,” “intuitive,” “didactic,” and “visual.” Some respondents also highlighted the importance of flexibility in using the tool in a time-convenient manner as well as being able to access from a mobile phone.

#### Level of Guidance and Social Connectedness

Regarding the level of guidance or support when using DMHTs, the majority of our sample reported preferring a guided intervention via a coach rather than a self-guided tool, expressing that the figure of a coach generates more confidence in the intervention and a greater sense of “closeness” despite the lack of in-person contact. Others mentioned the possibility of combining both modalities based on preference or demand. As for the profile of the coach, the most frequently mentioned professional role was a psychologist. However, rather than a professional role, most participants placed greater emphasis on the coach’s personal characteristics and skills, mainly highlighting empathy, active listening, receptivity, dynamism, and the ability to inspire confidence. As for the type of communication with the coach, the most preferred modality was videoconferencing, followed by telephone contact, and finally written messages. Some also mentioned the possibility of combining modalities, depending on the content and personal needs. Most of the participants preferred to accessing the content of the tool in stages or organized in modules rather than having it all available from the beginning. They indicated that this organization in modules would increase learnability by helping them navigate the tool in a more structured manner. Regarding content, some participants spontaneously emphasized the importance of having specific mental health content tailored to their needs rather than broad or general information. In addition, most noted that a reminder system, such as alarms, would be useful to aid engagement. Finally, regarding the possibility of being able to communicate with other users of the tool, a majority expressed that it would be a good idea, while a smaller group had doubts, and a few stated they would not use this option even if available.

#### Privacy and Confidentiality

Most participants reported that they would trust confidentiality protection mainly on the premise that when such an intervention is implemented within the context of a research study and with institutional backing, confidentiality should already be guaranteed. Among the few who expressed concerns, these were related to data privacy (sharing data with third parties), mentioning their concern about a possible link with social networks and the use of private data for advertising. On the other hand, almost all participants preferred nonanonymity with the figure of the coach to further “humanize” the intervention and increase confidence.

## Discussion

### Principal Findings

Our results showed that the worst stage of the pandemic in terms of mental health was the outbreak, with almost all workers experiencing some form of emotional distress. The main emotions that participants expressed during this stage, as well as their determinants, are consistent with those reported by other national and international studies conducted on frontline health care workers during the first wave of the pandemic [3-5,19-22].

Importantly, our results showed that perceived emotional distress, main emotions, and their determinants changed over time. We found that almost half of the sample reported an improvement in their emotional discomfort over time, expressing hope and relief. This improvement was associated with a greater feeling of control over the situation, confidence in vaccines, decreased measures of isolation, and the feeling of regaining prepandemic working dynamics. Nonetheless, more than 2 years after the outbreak, emotional distress persisted for over half of the sample. Fatigue and nervousness were the main expressed emotions, which were determined by a high workload, the feeling that pandemic times are not over, and sustained poor working conditions. This result is consistent with other epidemiological studies, which showed that poor mental health outcomes among health care workers tend to persist over time [23-25] and that the main symptoms of poor mental health tend to change with the different COVID-19 stages as well [26].

Fatigue, nervousness, and emotional exhaustion, which may occur in response to chronic work stressors, are common signs of burnout [27,28]. Fatigue, which is not only linked to lower job satisfaction and increased rates of absenteeism [29], can also impair concentration and slow reaction times, elevating the risk of workplace accidents or fatigue-related incidents [30]. The assessment of the determinants of such emotional distress provides valuable input into potential intervention strategies to respond to such calls. Multifaceted interventions with a holistic approach and implemented in a timely manner are needed to protect the mental health and well-being of these workers during and after the pandemic. Similar to recommendations and claims made for the health care workforce, interventions for care workers should also be targeted at the organizational or institutional level via systematic support and at the individual level by implementing psychological interventions for those workers with emotional distress [29,31,32]. It is not enough that the institutions have been reactive to the pandemic with concrete measures to deal with a sudden crisis. They need to implement long-lasting workplace changes to improve working conditions and promote the long-term well-being of their staff. At the individual level, offering tailored digital psychological interventions using cognitive behavioral therapy is a promising option for workers experiencing emotional distress [29].

Since digital mental health interventions can represent a promising individual-level strategy to improve the mental health of LTCWs, we identified several barriers and facilitators for their successful engagement in the context of the COVID-19 remission phase.

Not only did almost all participants deny having any previous experience in the use of DMHTs, but almost a half of them also spontaneously referred to their lack of skills in the use of digital tools. This is a potential barrier that may be linked to the mean age of the sample (44, SD 11.4 years), since previous studies found that younger people have higher adherence to this type of intervention than older people [33,34]. Despite this lack of experience, the vast majority of participants reported believing that using DMHTs could be helpful in improving their mental health. This is an important facilitator since people’s expectations and preconceived beliefs about whether a tool will be effective positively influence experience and engagement [35-37]. In this regard, addressing the expectations and beliefs of the target population before developing a DMHT (or as an early step during the development process) could help identify and consequently manage early barriers [37].

Most participants highlighted the importance of accessibility when using a DMHT. Similar to previous studies [9,37], we found that achieving “user-friendly” tools is a key facilitator for engagement. In addition, participants also highlighted the importance of flexibility, which would enable them to use the tool according to their needs and changing working shifts*.*

Regarding the level of guidance or support, the majority of participants reported a preference for a guided intervention with a human coach rather than a self-guided tool, which is also in line with findings from previous studies [11,37]. Increased support may enhance and extend engagement in DMHTs. In the context of a pandemic, where social contact may be limited, this feature becomes even more relevant. Reminder systems are also important facilitators since they could prevent forgetfulness and encourage users to stay committed [37]. Furthermore, satisfaction with the type of content and the manner the materials are offered is critical to engagement. Interestingly, most of our participants express a preference for the tool’s content to be delivered in stages or modules, aiming to enhance learnability and facilitate structured navigation. Additionally, some participants underscore the significance of receiving personalized content that fits their needs or interests. Therefore, addressing user needs is key to preventing dropouts and ensuring sustained engagement [9]. In turn, most of our sample expressed that having the possibility to communicate with other users of the tool would be beneficial. Prior to the pandemic, social connectedness through DMHTs had proven to be not only a facilitator for engagement [38] but also a tool with therapeutic value per se [39]. In the current context, where social interactions have changed, this possibility could be even more relevant.

Finally, most participants in our study reported a high level of trust in confidentiality. This places significant responsibility on those developing mental health interventions through DMHTs. Research has indicated that the transmission of data to third parties by mental health smartphone apps is prevalent, thereby denying users an informed choice regarding whether or not to accept such sharing [40]. This issue may be a consequence of most DMHTs existing outside of health care regulation and also falling outside of health care privacy legislation [9]. A clear and transparent written privacy policy should always be available to inform users about how their data are going to be used.

At this juncture, it is crucial to contemplate the transferability of our findings to other work environments and populations. Although our research focused on a specific group in a particular context, the emerging themes and shared emotional experiences might resonate in similar situations. The fluctuation in perceptions over time provides a dynamic perspective that can be insightful for understanding long-term impacts in the context of future global pandemics. On the other hand, the impact of variables such as age or digital skills, perceived as potential barriers to the adoption of DMHTs, could be extrapolated to other populations, such as informal carers. The preference for guided interventions and the significance of accessibility and flexibility, among other variables, are also elements that might have broader applications in the design of DMHTs. It is imperative to acknowledge that each work environment and demographic group has its own unique characteristics, and the direct application of our results may necessitate adjustments. Nevertheless, by presenting these findings in a detailed and transparent manner, we aim to provide valuable insights that can guide future research and intervention strategies in diverse settings.

### Limitations

This study acknowledges certain limitations that are crucial for contextualizing and evaluating the robustness of the findings. First, we did not account for participants’ preexisting mental health conditions (prior to COVID-19) when we assessed mental health needs. This could bear significant implications, as individuals with preexisting mental disorders are particularly vulnerable to the mental health threat of the pandemic. Second, the uneven gender distribution in our sample, with only 4 men, poses a limitation in terms of generalizing findings to this group. While our sample was not centralized in a single location, it is worth noting that all participating centers are located in the metropolitan area of Barcelona, which may limit the generalizability of our findings to the broader Catalonia region. Future studies should use more heterogeneous samples. Finally, inherent in qualitative research, there may be a potential for selection bias since workers were to some extent self-selected.

### Conclusions

We identified that the worst stage of the pandemic in terms of LTCWs’ mental health was the COVID-19 outbreak, in which all workers reported having experienced some form of emotional distress. We also identified a persistence of emotional distress over time in more than a half of the participants, with fatigue and nervousness being the most frequently expressed emotions. This suggests that, although many workers significantly improved their psychological discomfort, mental health problems in this group are still relevant even more than 2 years after the pandemic outbreak. Future studies are needed to determine the factors that promote or hinder resilience among this underrepresented population group in order to shape implementation strategies to promote well-being. Finally, this study also identified new barriers and facilitators to engagement with DMHTs during the remission phase of the COVID-19 pandemic. These findings provide key information for the development of tailored digital mental health interventions among LTCWs.

## Appendix

Multimedia Appendix 1 Interview guide.

Multimedia Appendix 2 Rapid Research Evaluation and Appraisal Lab Sheet.

## Abbreviations

COREQ: Consolidated Criteria for Reporting Qualitative Research
DMHT: digital mental health tool
LTCW: long-term care worker
RREAL: Rapid Research Evaluation and Appraisal Lab
REPICAL: reducing the psychosocial impact of the Covid-19 pandemic on workers of assisted living facilities

## Data Availability

The data sets generated during and analyzed during this study are available from the corresponding author on reasonable request.
