# Supplementary material for: Long-Term Emotional Impact of the COVID-19 Pandemic and Barriers and Facilitators to Digital Mental Health Tools in Long-Term Care Workers: Qualitative Study
Source: J Med Internet Res. 2024 May 29;26:e47546. doi: 10.2196/47546 (PMC11170039; doi:10.2196/47546)
Supplement: Multimedia Appendix 1 [file jmir_v26i1e47546_app1.pdf]

Appendix 1: Guía entrevista cualitativa – Proyecto REPICAL

Entrevistador/a:

Fecha:

Código ID de entrevistado/a:

Hora de inicio:

Hora de fin:

INTRODUCCIÓN

➞ Saludo

➞ Presentación

Mi nombre es ..., soy ...(formación)... y formo parte del grupo de investigación del Proyecto Repical, de la Fundació Sant Joan de Déu.

➞ Agradecimiento

Primero que nada, quiero agradecerte, en nombre de todo el equipo investigador, por tu participación en el estudio y por tu tiempo.

➞ Objetivos y etapas de la entrevista

Como te hemos contado en la hoja informativa, los objetivos que nos proponemos con esta entrevista son:

- *Conocer el impacto de la pandemia por COVID-19 en los trabajadores de residencias geriátricas y centros socio-sanitarios, así como las principales necesidades del colectivo.*
- *Conocer su opinión sobre la estrategia de intervención que estamos desarrollando.*

En base a estos datos, adaptaremos la intervención para la FASE 2 del proyecto.

La entrevista constará de tres etapas:

- *Primero te realizaré algunas preguntas generales sobre ti y tu actividad laboral.*
- *Luego, hablaremos sobre las que han sido tus vivencias durante la pandemia y como te has sentido durante la misma, para lo que también te iré guiando con preguntas.*
- *Por último, te preguntaré por tu opinión sobre distintos aspectos de la estrategia que estamos desarrollando para la fase 2 del estudio.*

➞ Confidencialidad y modalidad de la entrevista

Antes de comenzar quiero informarte que todo lo que hablemos en esta entrevista es confidencial.

Como ya te hemos comentado, el audio de la entrevista será gravado. Esto es para luego poder transcribir los datos.

Si hay alguna pregunta durante la entrevista que te haga sentir incómoda/o o que no quieras contestar por cualquier otro motivo, no dudes en decírmelo, no habrá ningún problema.

➞ Despejar dudas

¿Te ha quedado alguna duda o hay algo que quieras preguntarme antes de iniciar?

SECCIÓN 1. ASPECTOS SOCIO-DEMOGRÁFICOS

1. Edad:
2. Género:
3. Nacionalidad: española o extranjera
4. Máximo nivel de educación formal alcanzado:
5. Estructura familiar (composición del hogar):
    a. Vive sola/a
    b. Compañero/a sentimental
    c. Hijos/as
    d. Hijos/as + compañero/a sentimental
    e. Hijos/as + compañero/a sentimental + padres o suegros
    f. Otros parientes
    g. Amigos
    h. Otros:
6. Rol o posición en su trabajo:
7. Años de experiencia profesional en el rubro (en total):
8. Modalidad de trabajo actual:
    a. Tiempo completo
    b. Tiempo parcial
    c. Baja por enfermedad
9. Clasificación por color actual de la institución:

    Verde: centros sin casos activos de COVID-19 y correctamente sectorizados.

    Naranja: centros con casos activos de COVID-19 pero bien sectorizados y con brote controlado.

    Rojo: situación no controlada, sea por la dificultad de sectorización de los espacios o por la aparición de nuevos casos.

SECCIÓN 2. IMPACTO DE LA PANDEMIA POR COVID-19 EN LA SALUD MENTAL

Ahora te realizaré las preguntas sobre tus vivencias durante la pandemia por COVID-19 y su posible influencia sobre tu salud emocional.

10. *¿Cuál consideras que ha sido la peor etapa (o momento) de la pandemia para tu salud emocional?*
11. *¿Cómo te has sentido en esa etapa/momento?*
12. *¿Cuáles crees que han sido las causas más frecuentes de esos sentimientos?*
13. *¿Has hecho algo para abordar este malestar?*

    ¿Qué cosas?
14. *¿Consideraste en algún momento consultar por algún tipo de apoyo/ayuda psicológico para lidiar con la situación?*
    a. Si negativo: *¿por qué no lo has considerado?*
    b. Si afirmativo: *¿has podido acceder?*
        i. Si afirmativo: *¿a qué tipo de asistencia en salud mental has accedido (presencial, telefónico, online, etc.)?*
        ¿Cómo calificarías la experiencia?
        ii. Si negativo: *¿podrías describirme con qué barreras te has encontrado para el acceso?*
15. *¿Actualmente cómo te estás sintiendo emocionalmente?*
16. *¿Cuáles crees que son las causas más frecuentes de estos sentimientos?*

SECTION 3. SALUD MENTAL DIGITAL

** constructos basados en el marco de trabajo desarrollado por Borghouts et al., J Med Internet Res 2021, vol 23, iss 3, e24387.*

Constructo: creencias

1. *¿Alguna vez has usado herramientas digitales en salud mental?*

(definición: tecnología digital para evaluación, apoyo, prevención y tratamiento en salud mental, como apps o recursos webs)

Si afirmativo: *¿Cómo calificarías la experiencia?*

Si negativo: *¿Qué piensas respecto al uso de estas herramientas?*

2. *¿Crees que estas herramientas podrían ser útiles para mejorar tu salud mental?*

Estamos desarrollando una intervención en salud mental en formato digital (web/app), y nos gustaría saber tu opinión respecto a varios aspectos:

Constructo: Integración en la vida diaria

3. *¿Qué características crees que debería tener una intervención de este tipo para que te resulte fácil de integrar a tu día a día?*

   Ejemplo (si no hay respuesta espontánea): *¿Haces uso de alguna herramienta digital que te resulte fácil de integrar? ¿Qué características de la herramienta te lo facilitan?*

Constructo: nivel de orientación y de conexión social

4. *Si utilizaras una herramienta digital en salud mental, como una app para el móvil, ¿qué tipo de soporte te gustaría tener?*

   Nota: primero dejar al participante desarrollar su respuesta. Luego, preguntar específicamente sobre estos ítems:

   - *En cuanto a nivel de guía o apoyo: ¿preferirías que fuera auto-guiada (solo con recordatorios) o con apoyo (guía o facilitador)?*
   - *Si fuera auto-guiada:*

     ¿qué tipo de recordatorios quisieras que tuviera?

     ¿Con qué frecuencia?
   - *Si fuera con apoyo:*

     ¿qué tipo de apoyo preferirías (llamada telefónica, cara a cara, mensajes por escrito)?

     ¿La comunicación con el facilitador, la preferirías en sincrónico (ej.: chat) o en diferido?

     ¿Cuál te gustaría que fuera el perfil del facilitador o guía?
   - *¿Preferirías que el contenido fuera estando disponible por etapas (por ej.: cada semana un nuevo módulo) o poder verlo todo desde el inicio?*

- *¿Te gustaría poder comunicarte con otros usuarios de la herramienta (pares)?*

Constructo: privacidad y confidencialidad

5. *¿Qué podrías decirnos sobre la privacidad y confidencialidad a la hora de usar herramientas digitales en salud mental?*

   (por ej., en términos de: almacenamiento de datos, anonimato tuyo y/o del facilitador, compartir información con el facilitador, privacidad del ambiente físico)

CIERRE

¿Hay alguna cosa más que quieras compartir con nosotros y que yo no te haya preguntado?

Gracias por tu tiempo y por compartir tus opiniones y experiencias con nosotros.
