# Supplementary material for: Long-Term Emotional Impact of the COVID-19 Pandemic and Barriers and Facilitators to Digital Mental Health Tools in Long-Term Care Workers: Qualitative Study
Source: J Med Internet Res. 2024 May 29;26:e47546. doi: 10.2196/47546 (PMC11170039; doi:10.2196/47546)
Supplement: Multimedia Appendix 2 [file jmir_v26i1e47546_app2.pdf]

Appendix 2: RREAL Sheet

Participantes	SECCIÓN 1								
	Edad	Género	Nacionalidad (ESP/extranjera)	Composición del hogar:<br>- Solo<br>- Pareja sentimental<br>- Hijos/as<br>- Hijos/as + pareja sentimental<br>- Otros parientes<br>- Amigos<br>- Otros:	Máximo nivel de educación formal alcanzado	Rol o posición en su trabajo	Años de experiencia profesional (en total)	Modalidad de trabajo actual:<br>- Tiempo completo<br>- Tiempo parcial<br>- Baja por enfermedad	Código de clasificación por color en la institución: (verde, naranja, rojo)
REP-001									
REP-002									
REP-003									
REP-004									

SECCIÓN 2

Etapa de la pandemia que considera como la peor para su salud emocional	Sentimientos referidos en dicha etapa	Causas identificadas como las más frecuentes del malestar	¿Utilizó alguna estrategia para abordar el malestar? ¿Cuál/es?	¿Consideró consultar por algún tipo de apoyo/ayuda psicológico para lidiar con la situación?	Si no lo consideró: motivos expresados

Si lo consideró: ¿ha podido acceder?	Si ha accedido: ¿a qué tipo de asistencia en salud mental ha accedido? (presencial, telefónico, online, etc.)	¿Cómo califica la experiencia?	Si no ha accedido: barreras encontradas para el acceso	Estado emocional actual	Causas más frecuentes identificadas del estado emocional actual

SECCIÓN 3

¿Usó alguna vez herramientas digitales en salud mental?	Si utilizó: ¿Cómo califica la experiencia?	Si no utilizó: opinión respecto al uso de herramientas digitales en salud mental	¿Cree que estas herramientas podrían ser útiles para mejorar su salud mental?	Características que cree que debería tener la intervención para que le resulte fácil de integrar a su día a día	Si utilizara una herramienta digital en salud mental, ¿qué tipo de soporte le gustaría tener? (respuesta libre)

Preferencia en nivel de guía o apoyo: auto-guiada (solo con recordatorios) o apoyo (facilitador).	Tipo de recordatorios de preferencia	Frecuencia de recordatorios de preferencia	Tipo de comunicación de preferencia con el facilitador (llamada telefónica, cara a cara, mensajes de texto, etc)	Timing de preferencia para la comunicación: en sincrónico (ej.: chat) o en diferido	¿Cuál le gustaría que fuera el perfil del facilitador o guía?

**Preferencia en la presentación del contenido: disponible por etapas (por ej.: cada semana un nuevo módulo) o todo disponible desde el inicio**	**¿Le gustaría poder comunicarse con otros usuarios de la herramienta?**	**¿Qué opina sobre la privacidad y confidencialidad en el uso de herramientas digitales en salud mental?**	**Otros comentarios**	**Misceláneos**
